# Asthma-prone areas modeling using a machine learning model

**DOI:** 10.1038/s41598-021-81147-1

**Published:** 2021-01-21

**Authors:** Seyed Vahid Razavi-Termeh, Abolghasem Sadeghi-Niaraki, Soo-Mi Choi

**Affiliations:** 1 Geoinformation Tech. Center of Excellence, Faculty of Geodesy and Geomatics Engineering, K.N. Toosi University of Technology, 19697 Tehran, Iran; 2 Departmet of Computer Science and Engineering, and Convergence Engineering for Intelligent Drone, Sejong University, Seoul, Republic of Korea

**Keywords:** Immunology, Environmental sciences, Gastroenterology, Risk factors, Diseases, Respiratory tract diseases

## Abstract

Nowadays, owing to population growth, increasing environmental pollution, and lifestyle changes, the number of asthmatics has significantly increased. Therefore, the purpose of our study was to determine the asthma-prone areas in Tehran, Iran considering environmental, spatial factors. Initially, we built a spatial database using 872 locations of children with asthma and 13 environmental factors affecting the disease—distance to parks and streets, rainfall, temperature, humidity, pressure, wind speed, particulate matter (PM 10 and PM 2.5), ozone (O_3_), sulfur dioxide (SO_2_), carbon monoxide (CO), and nitrogen dioxide (NO_2_). Subsequently, utilizing this spatial database, a random forest (RF) machine learning model, and a geographic information system, we prepared a map of asthma-prone areas. For modeling and validation, we deployed 70% and 30%, respectively, of the locations of children with asthma. The results of spatial autocorrelation and RF model showed that the criteria of distance to parks and streets as well as PM 2.5 and PM 10 had the greatest impact on asthma occurrence in the study area. Spatial autocorrelation analyses indicated that the distribution of asthma cases was not random. According to receiver operating characteristic results, the RF model had good accuracy (the area under the curve was 0.987 and 0.921, respectively, for training and testing data).

## Introduction

Today, with the growth of societies, diseases are increasing in terms of diversity and the number of people involved. One of the diseases that has become extremely common is asthma^1^. The immune system of people with asthma reacts more than usual to seemingly harmless substances in the habitat. The number of patients with asthma is increased by 5% every year^2^. Over the past 50 years, asthma has increased dramatically among children in modern and developed countries because of the contamination of the environment with stimulants^3^. According to the latest report of the World Health Organization (WHO), the number of asthmatics in the world is 300 million, which is estimated to increase to 400 million by 2025^4^. In 2015, about 82% of deaths in Iran were due to chronic noncommunicable diseases, of which 4% were related to respiratory diseases. The prevalence of asthma in Tehran province is higher than that in other provinces of Iran and is 12.6% in the age group 6–7 years old and 16.6% in the age group 13–14 years old. The higher prevalence of asthma in Tehran, compared to the general statistics of Iran, is due to various factors involved in asthma, including high air pollution in Tehran province^5^. Owing to the large number of asthma patients, if they were untreated and uncontrolled, it could lead to a serious problem for public health^5^.

There are several factors involved in the development and exacerbation of this disease, which vary depending on the type of geography, environmental conditions, and lifestyle of individuals. Identifying allergens and preventing exposure to allergens is the best way to prevent allergies^6^. Since an important part of these factors is related to the human environment, the discovery of environmental factors affecting the prevalence of asthma can play a significant role in reducing its effects. Therefore, by collecting appropriate information about the living environment of individuals, the role of various environmental factors in the occurrence and exacerbation of this disease can be measured^7^.

The technology of geographic information system (GIS) is particularly useful in assessing the relationship between disease occurrence and environmental quality. GIS can be applied to process health data, analyze geographical distribution, and prepare a disease prediction map, surveillance, and epidemic management. Location-based analysis can be effective in the conduct of epidemiological study of asthma risk factors (exposure), the identification of areas prone to asthma, and the prevention and management of the disease^8^. So far, many studies have implemented GIS to analyze asthma spatially. Hashimoto et al. investigated the effect of climate on emergency patients with asthma attacks in Tokyo, Japan^9^. According to their results, high pressure, humidity, and temperature have a significant positive correlation with asthma. Zanolin et al. found in several Italian cities that the prevalence of asthma and its symptoms increased with decreasing latitude and increasing average annual temperature^10^. Peled et al. proposed a spatial approach to asthma behavior in children in Israel^11^. Maantay proposed a GIS-based approach to investigate the relationship between air pollution and asthma in New York^12^. The results revealed that people living near harmful areas were 66% more likely to develop asthma. Ahmad Khan et al. examined the relationship between asthma and vegetation with a GIS-based approach in Karachi, Pakistan and proved a direct correlation^13^. Gorai et al. deployed a GIS-based approach to assess the relationship between air pollution and asthma in New York, USA^14^. According to the results, there is a significant relationship between particulate matter (PM) 2.5 and carbon dioxide and asthma. Chang et al. modeled the distribution of asthma with environmental variables^4^. They thereupon utilized regression to identify vulnerable blocks associated with asthma. Škarková et al. prepared an asthma distribution map using environmental factors and GIS^15^. For this purpose, data from 13,456 children with asthma as well as air pollution data (CO, O_3_, NO_2_, SO_2_, PM 2.5, and PM 10), traffic congestion, distance to street, land cover, and agricultural products used the study area. Douglas et al. examined environmental factors, asthma reports, diesel particles, and public parks in Los Angeles^16^. For this purpose, the spatial analysis of hot spots, least squares method, and weighted geographical regression were used to map high-risk areas for asthma. Prediction is the process of estimating unknown situations. Forecasting provides an estimation of future events and can turn past experiences into predicting future events^17^. Given that we face a large number of effective criteria and disease data in predicting areas prone to asthma, big data are discussed. A good tool for big data analysis is machine learning. The main purpose of machine learning is to better understand the data and discover the relationships between dependent and independent variables and ultimately estimate a value^18^. Although machine learning models require more observational data to learn, they are faster and more efficient than traditional methods and have fewer limitations by some assumptions^19^. Machine learning models often perform better in various areas of environmental research in terms of accuracy, speed, and computational cost^20^. Random forest (RF) is one of the machine learning models that has been considered in environmental modeling in recent years owing to its simplicity, robustness, and capacity to deal with complex data^21^. According to the authors’ knowledge, although the RF model has not been implemented to assess areas susceptible to asthma, its good performance has been proved in other environmental fields, such as groundwater potential^33^, groundwater hardness^22^, flood risk^23^, and PM 10 risk^19^.

Therefore, the purpose of this study was to map the areas prone to asthma using the RF model and environmental factors in Tehran, Iran. The innovation of the present study is the application of RF machine learning modeling in combination with GIS to determine asthma-prone areas by considering environmental factors affecting asthma.

## Methodology

This research was conducted in five steps. In the first step, a spatial database was created using the location of children with asthma and 13 environmental factors affecting asthma. In the second step, using the frequency ratio (FR) model, the spatial relationship between asthmatics and environmental factors was determined. In the third step, the spatial autocorrelation of asthma incidence was examined. In the fourth step, the RF model was deployed to determine the asthma-prone areas. In the last step, modeling was evaluated using the receiver operating characteristic (ROC) curve and sensitivity analysis.

### Study area

**Figure 1:**
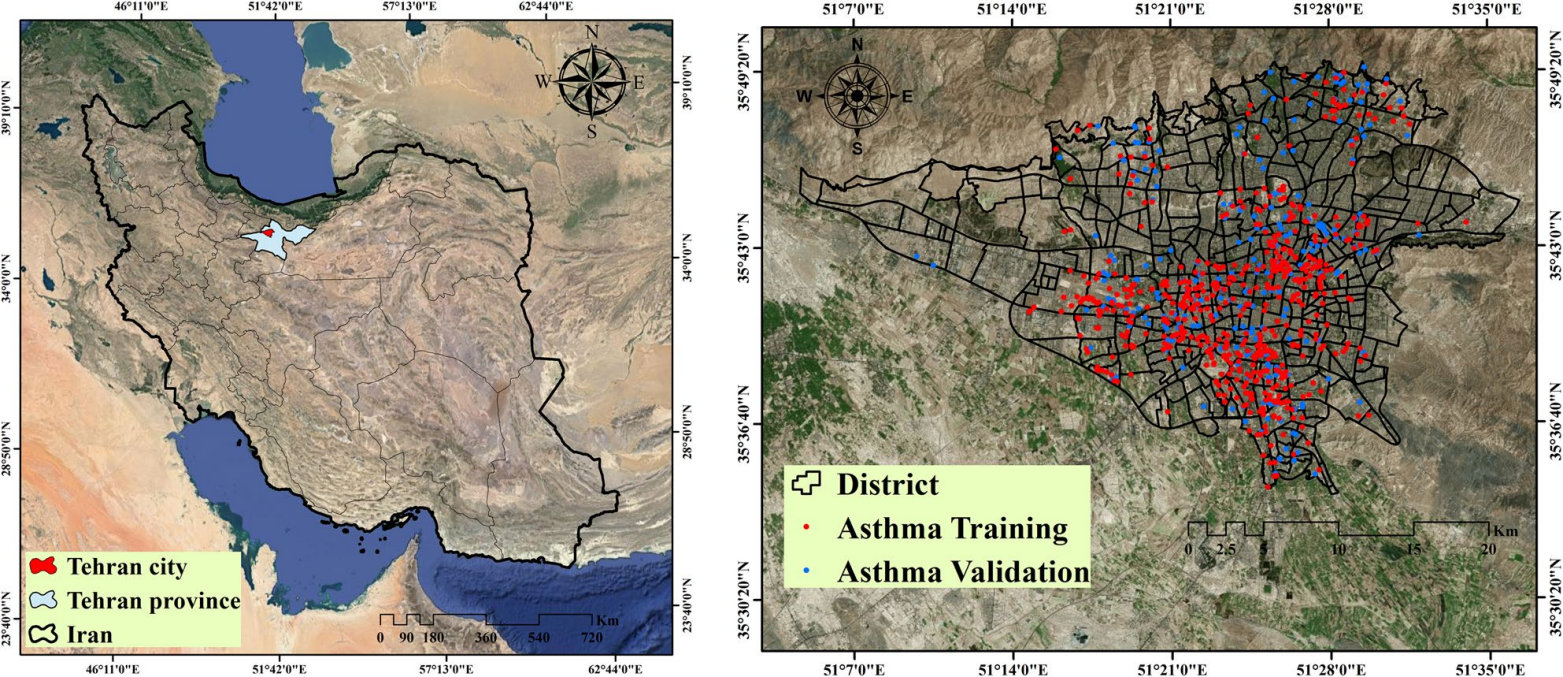
Study area with locations of asthma patients. This map was generated using the tool of ArcGIS 10.3 (ESRI, Redlands, CA, USA, http://www.esri.com).

Tehran is the capital city of Iran and has a population of 8,693,706. In terms of population, it is ranked first in West Asia and 24th globally. Tehran has an area of 730 km^2^ and is located at a longitude between 51°17′ E and 51° 33′ E and a latitude between 35° 36′ N and 35° 44′ N. Tehran’s altitude ranges from 900 to 1800 m above sea level; it decreases from north to south. Air pollution is one of the most important environmental problems in Tehran and derives from geographical factors, e.g., the enclosing effect of mountains, vehicles, e.g., cars and motorcycles, fuel houses, and pollution from factories. The location of Tehran is shown in Fig. 1.

### Spatial database

**Figure 2:**
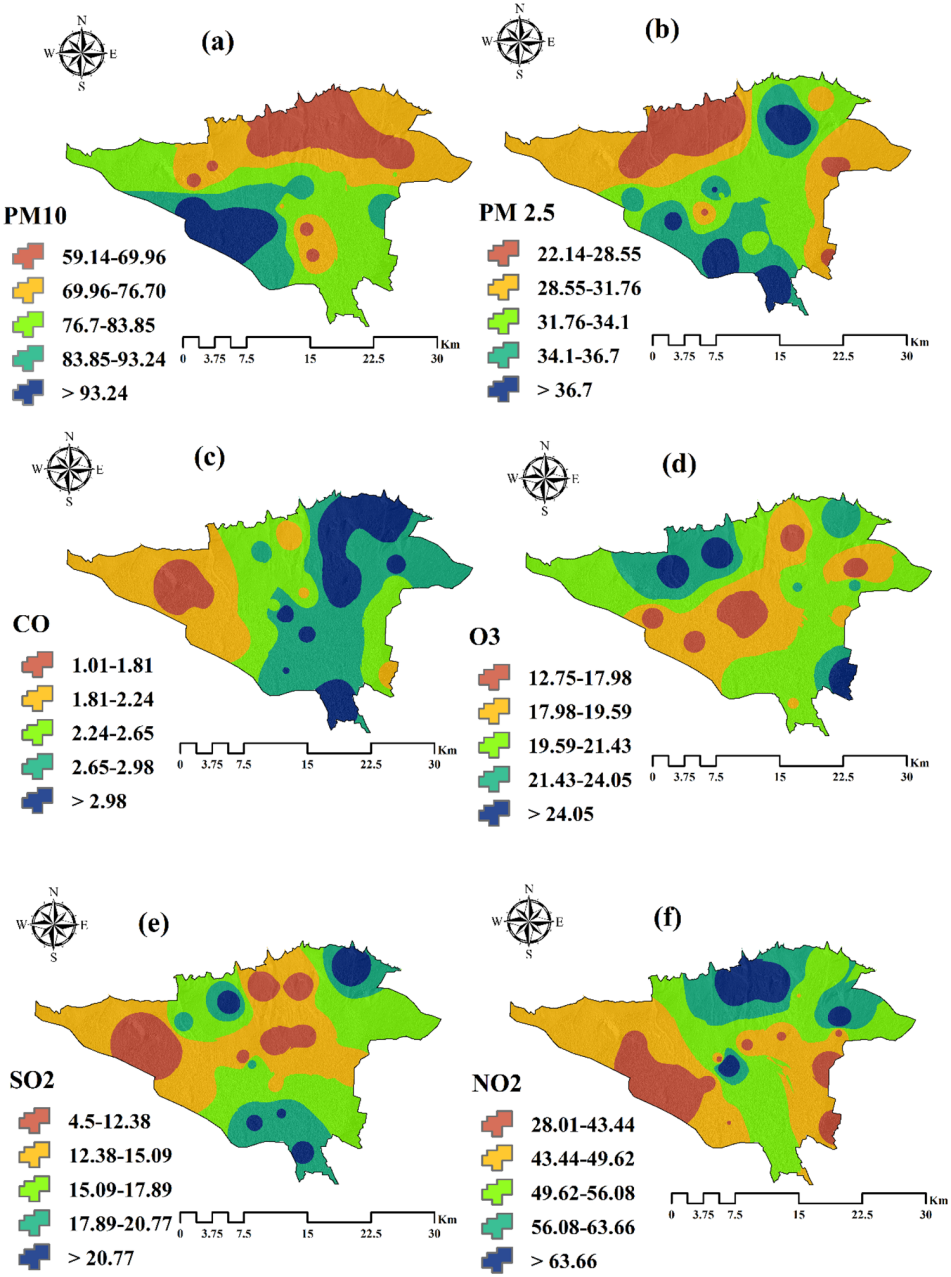

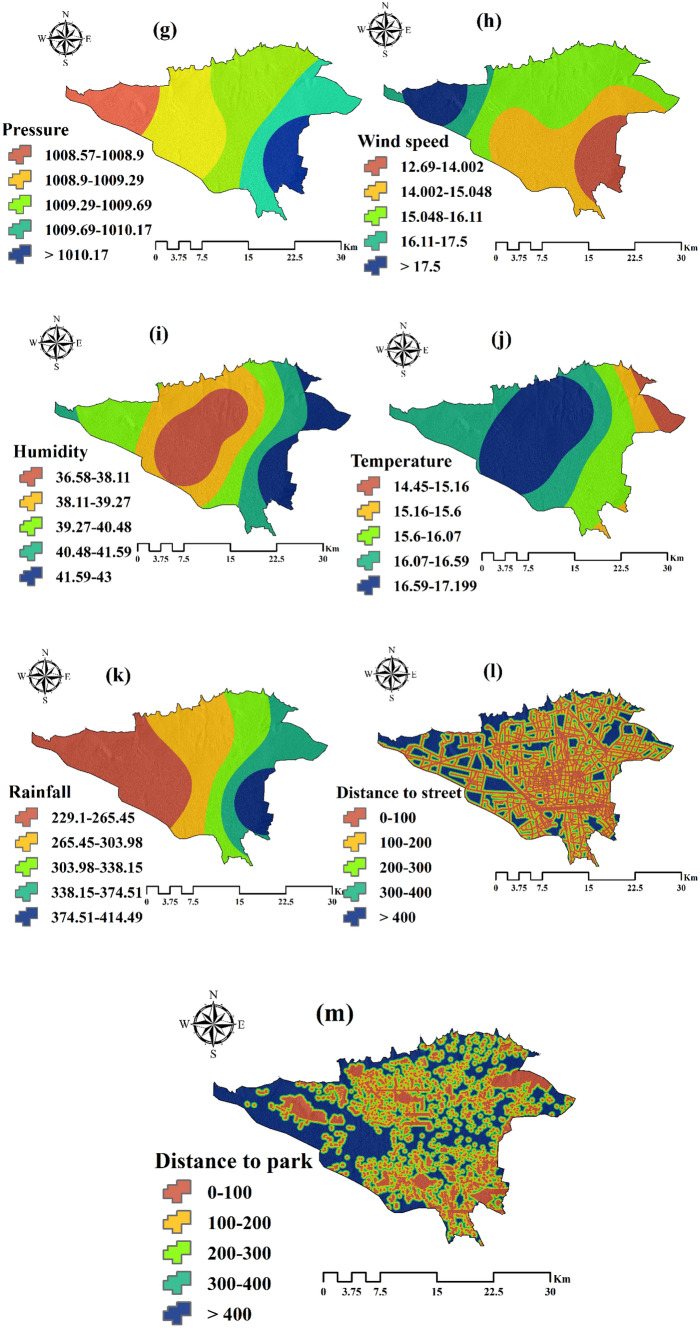
Environmental criteria affecting asthma. (**a**) Particulate matter PM 10, (**b**) PM 2.5, (**c**) CO, (**d**) O_3_, (**e**) SO_2_, (**f**) NO_2_, (**g**) Pressure, (**h**) Wind speed, (**i**) Humidity, (**j**) Temperature, (**k**) Rainfall, (**l**) Distance to street, and (**m**) Distance to park. This map was generated using the tool of ArcGIS 10.3 (ESRI, Redlands, CA, USA, http://www.esri.com).

In the first step, independent and dependent datasets were used to create a spatial database. Dependent data included the locations of asthmatic children in 2019 in Tehran. These data were obtained from the Hospital Information System, one of the largest centers for the provision of medical services in the field of respiratory diseases (872 cases). We used 70% (611 cases) of asthmatics’ position data for modeling and 30% (261 cases) for evaluation (see Fig. 1). According to WHO’s reports and previous research, environmental factors affecting asthma have been identified. These factors include air pollution parameters (O_3_, CO, NO_2_, SO_2_, PM 10, and PM 2.5), meteorological parameters (rainfall, temperature, humidity, pressure, and wind speed), distance to streets, and distance to parks. Air pollution data were prepared using 23 pollution measuring stations of the Tehran Air Pollution Control Company. For this purpose, the annual average of these parameters in the period 2009–2019 was used. To prepare a map of meteorological parameters, the annual average of these parameters was used for 12 meteorological stations in Tehran province from 2009 to 2019. The Kriging interpolation method was applied in ArcGIS 10.3 environment to map the air pollution and meteorological parameters. Criteria for distance to street and distance to park were prepared using the land use map of Tehran. Environmental factors affecting asthma are shown in Fig. 2.

### Spatial autocorrelation analysis

In environmental studies, there are often data that are not independent, and their dependence is due to their locations in the study space^24^. The main assumption of most common statistical methods is based on data independence. Therefore, owing to the correlation and spatial effect between these types of data, this assumption is not actually realized and the data are interdependent, thereby conventional statistical methods are not suitable for studying them^25^. Hence, geostatistical methods are the suitable option. To model events such as diseases, we need first examine the spatial autocorrelation between their occurrences and determine which distribution (random, dispersed, or cluster) follows the spatial pattern of the event in the region^26^. In spatial autocorrelation, there are two approaches including the spatial structure and structural function. In spatial structure, the spatial pattern of the data is studied. Here, we utilized Moran’s I and Getis-Ord’s indexes for this purpose. In the structural function, the spatial dependence of the data is addressed; it uses semivariance to measure the spatial dependence between two observations as a function of the distance between them. Semivariogram is a graph of how semivariance changes as the distance between observations changes^27^.

### Moran’s I index

This index is one of the tools to study spatial autocorrelation between spatial data. In a dataset, the Moran’s I is between − 1 and + 1. If the Moran's I index value is higher than zero, the spatial autocorrelation is positive; if it is lower than zero, it is negative; and if it is close to zero, no spatial autocorrelation exists^28^. The Moran’s I index is calculated using Eq. (1):

1 $$I=\left(\frac{N}{{\sum }_{i=1}^{N}{\sum }_{j=1}^{N}{w}_{ij}}\right)*(\frac{{\sum }_{i=1}^{N}{\sum }_{j=1}^{N}{w}_{ij}({x}_{i}-x)({x}_{j}-x)}{{\sum }_{i=1}^{N}{({x}_{i}-x)}^{2}})$$

where ${x}_{i}$ and ${x}_{j}$ are the numbers of asthma cases in polygon i and j, respectively, x is the average number of asthma cases, N is the total number of asthma cases, and ${w}_{ij}$ is the spatial weight between polygons i and j.

In local Moran’s I index, this analysis investigates the relation between points and neighbors, in which four cases might occur^28^:

- High–High (H–H): when both the spatial autocorrelation of that value and its neighbors are positive.
- High–Low (H–L): when the former is positive and the latter negative.
- Low–High (L–H): when the former is negative and the latter positive.
- Low–Low (L–L): when both the former and latter are negative.

### Getis-Ord Gi* index

This index is used to examine the accumulation of very large or very small amounts of the occurrence of an event, which includes indicators of hot spots (high-risk areas) and cold spots (low-risk areas). Positive Z-score values indicate hot spots and negative Z-score values indicate cold spots^29^. The Getis-Ord Gi* index is calculated from Eq. (2):

2 $$G=\left(\frac{{\sum }_{i=1}^{N}{\sum }_{j=1}^{N}{w}_{ij}{x}_{i}{x}_{j}}{{\sum }_{i=1}^{N}{\sum }_{j=1}^{N}{x}_{i}{x}_{j}}\right)$$

where ${x}_{i}$ and ${x}_{j}$ are the numbers of asthma cases in polygon i and j, respectively, N is the total number of asthma cases, and ${w}_{ij}$ is the spatial weight between polygons i and j.

### Semivariogram

Semivariogram is known to detect the spatial coherence of a variable. Spatial coherence means that adjacent specimens are interdependent to a certain distance, and it is assumed that the dependence between specimens can be represented by a mathematical model called semivariogram^30^. Semivariogram is calculated using Eq. (3).

3 $$\gamma \left(h\right)=\frac{1}{2N\left(h\right)}{\sum }_{i=1}^{N\left(h\right)}{\left[Z\left({x}_{i}\right)-Z\left({x}_{i}+h\right)\right]}^{2}$$

where h denotes the distance in the specified direction between the position ${x}_{i}$ and ${x}_{i}+h$, N(h) the number of pairs of samples at a distance h from each other, $\gamma \left(h\right)$ the value of semivariogram for distance h, $Z\left({x}_{i}\right)$ the sample value at point ${x}_{i}$, and $Z\left({x}_{i}+h\right)$ the sample value at point ${x}_{i}+h$.

Semivariogram has three parameters—range, sill, and nugget—which are defined as follows^31^:

- Range (or radius of impact): It is the distance at which the variogram reaches a fixed point and approaches the horizontal line.
- Sill: It is the constant value the variogram reaches in the range of effect. Its value equals to the total variance of all the samples used to calculate the facade change.
- Nugget: It is the value of the variogram at the origin, i.e., for h = 0. Ideally, its value should be zero.

To determine the best correlation, the spatial dependence index based on Eq. (4) is used.

4 $$SD=\frac{nugget }{nugget+ partial sill}\times 100$$

Its value is examined in three cases: if it is less than 25%, it means strong spatial correlation; between 25 and 75%, moderate spatial correlation; and more than 75%, weak spatial correlation^32^.

### The FR model

In the FR model, the set of training points are introduced as a dependent variable, whereas the parameters affecting asthma are introduced as independent variables^33^. This model calculates the probability of the occurrence of asthma in each class for all criteria. To determine the effect of each class, each variable independent of Eq. (5) is used^34^.

5 $$FR=\frac{{\mathrm{F}}_{i}}{{P}_{i}}$$

where FR is the effect of each class of each parameter, ${\mathrm{F}}_{i}$ the percentage of training points located in class i, and ${P}_{i}$ the percentage of the pixels of class i in the entire study area.

### The RF model

The RF model was proposed by Breiman as a cumulative learning method for regression and clustering problems based on decision tree development^35^. An RF is a collection of trees not pruned, which are obtained with a recursive segmentation algorithm^36^. An RF is constructed using a set of trees based on N independent observational data. This model is a combination of several decision trees in which several bootstrap instances of the data are involved and a number of input variables are randomly involved in the construction of each tree. Using the bootstrap method, a large number of N samples from the primary observational datasets are sampled and placed. About one third of the data is not sampled during the sampling process and is considered an out-of-process sample. After constructing all the trees, the test data are introduced to the tree, and the number of trees for the input vector of output is obtained. By averaging these outputs, the final output is calculated^33^.

### Validation

Here, to evaluate the modeling of asthma-prone areas, the ROC index and area under the curve (AUC), root mean square error (RMSE) and mean absolute error (MAE), and sensitivity analysis were used.

#### ROC curve

The ROC curve consists of two axes of sensitivity (x-axis) and a transparency axis (y-axis). These axes are calculated through Eqs. (6) and (7), which are obtained from the comparison matrix with the definition of the threshold between zero and one^37^.

6 $$\mathrm{X}=1-\left[\frac{\mathrm{TN}}{\mathrm{TN}+\mathrm{FP}}\right]$$

7 $$Y=\left[\frac{\mathrm{TP}}{\mathrm{TP}+\mathrm{FN}}\right]$$

where TP denotes the pixels that are correctly assigned to the desired category, TN the pixels that are not properly assigned to the category, FP the pixels that are incorrectly assigned to the desired category, and FN the pixels that are not incorrectly assigned to the desired category^33^.

The area below the ROC curve is called AUC. Its value varies between 0.5 and 1; the closer it is to one, the higher the modeling efficiency is^34^.

#### RMSE and MAE indexes

Predictive error, as a quantitative method, defines the difference between observed and estimated values, which is used to determine the accuracy of the model. Here, to evaluate the modeling accuracy, RMSE and MAE indices were used in the form of Eqs. (8) and (9)^33^.

8 $$RMSE=\sqrt{\frac{{\sum }_{i=1}^{n}{({y}_{i}-\stackrel{-}{{y}_{i}})}^{2}}{N}}$$

9 $$MAE=\frac{{\sum }_{i=1}^{n}\left|({y}_{i}-\stackrel{-}{{y}_{i}})\right|}{N}$$

where N denotes the total number of training data, ${y}_{i}$ the observed values, and $\stackrel{-}{{y}_{i}}$ the predicted values.

#### Sensitivity analysis

Sensitivity analysis shows how modeling input’s changes affect modeling output. By eliminating any of the effective criteria, the necessity of their presence or absence is determined^38^. Sensitivity analysis is conducted using Eq. (10).

10 $$RD=\frac{{AUC}_{all}-{AUC}_{i}}{{AUC}_{all}}*100$$

where RD is the relative decrease index, ${AUC}_{all}$ the final AUC value of the training data for all parameters, and ${AUC}_{i}$ the AUC value for the training data where parameter i is omitted^39^.

## Results

### Spatial autocorrelation result

**Figure 3:**
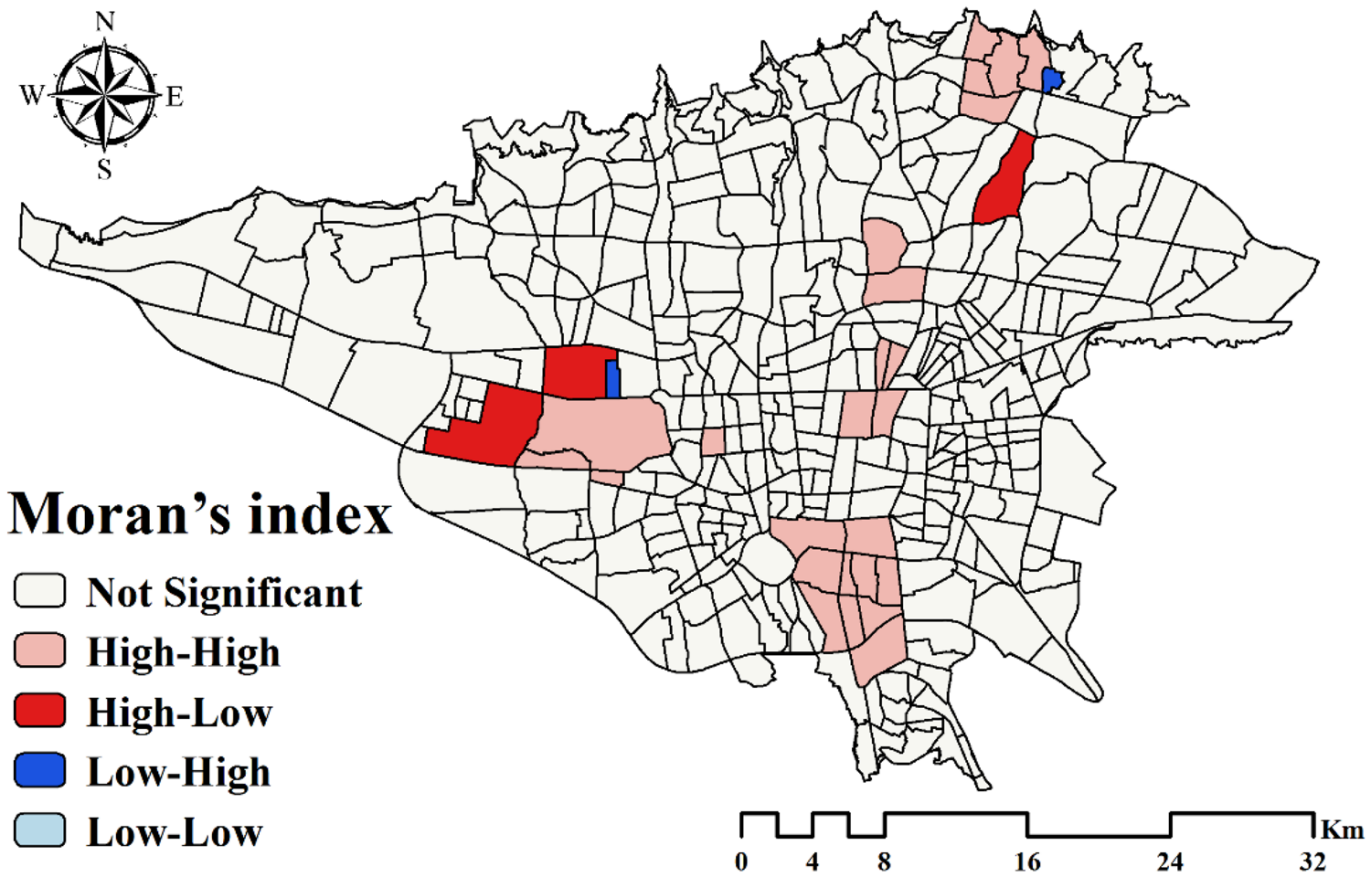
Spatial clusters using Moran’s I index. This map was generated using the tool of ArcGIS 10.3 (ESRI, Redlands, CA, USA, http://www.esri.com).

**Figure 4:**
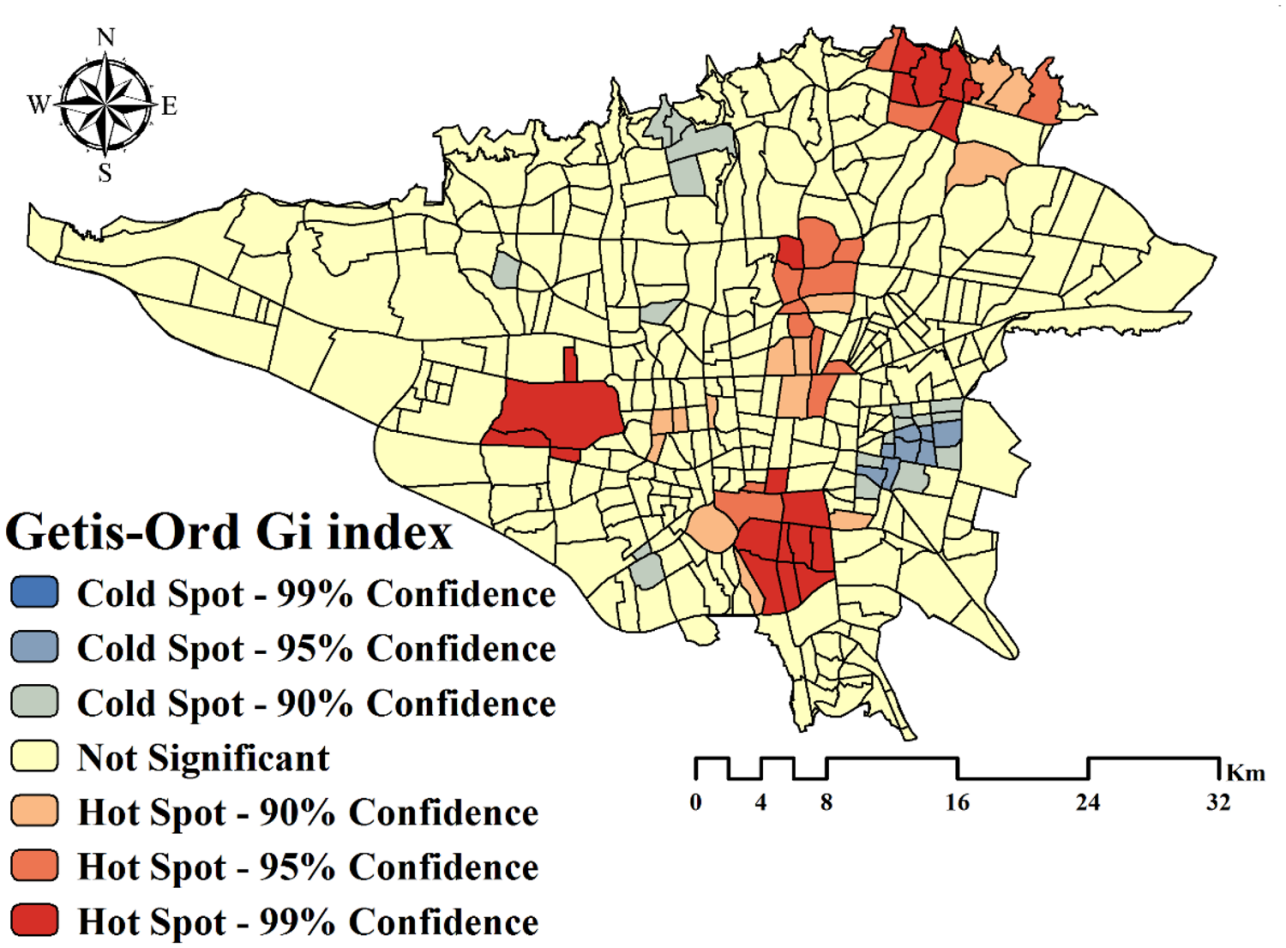
Spatial clusters using Getis-Ord Gi* index. This map was generated using the tool of ArcGIS 10.3 (ESRI, Redlands, CA, USA, http://www.esri.com).

The results for the Moran’s I and Getis-Ord Gi* indexes are presented in Table 1. According to them, the distribution of asthma in the study area was clustered. P-value parameter is small and shows that the results of autocorrelation tests are statistically significant, the condition of the null hypothesis is correct based on the observed data, and the distribution of disease is not random. Spatial clusters using Moran’s I and Getis-Ord Gi* indexes are shown in Figs. 3 and 4, respectively. The high-high and hot spot areas indicate areas where disease clusters are present. The low-low and cold spot areas indicate areas where disease-free clusters are present.

**Table 1:** Results of the spatial autocorrelation indexes.

Index	Index value	z-score	p-value	Distribution type
Moran’s I	0.149	6.807	0.00	Clustered
Getis-Ord Gi*	0.000029	2.673205	0.007513	Clustered

**Figure 5:**
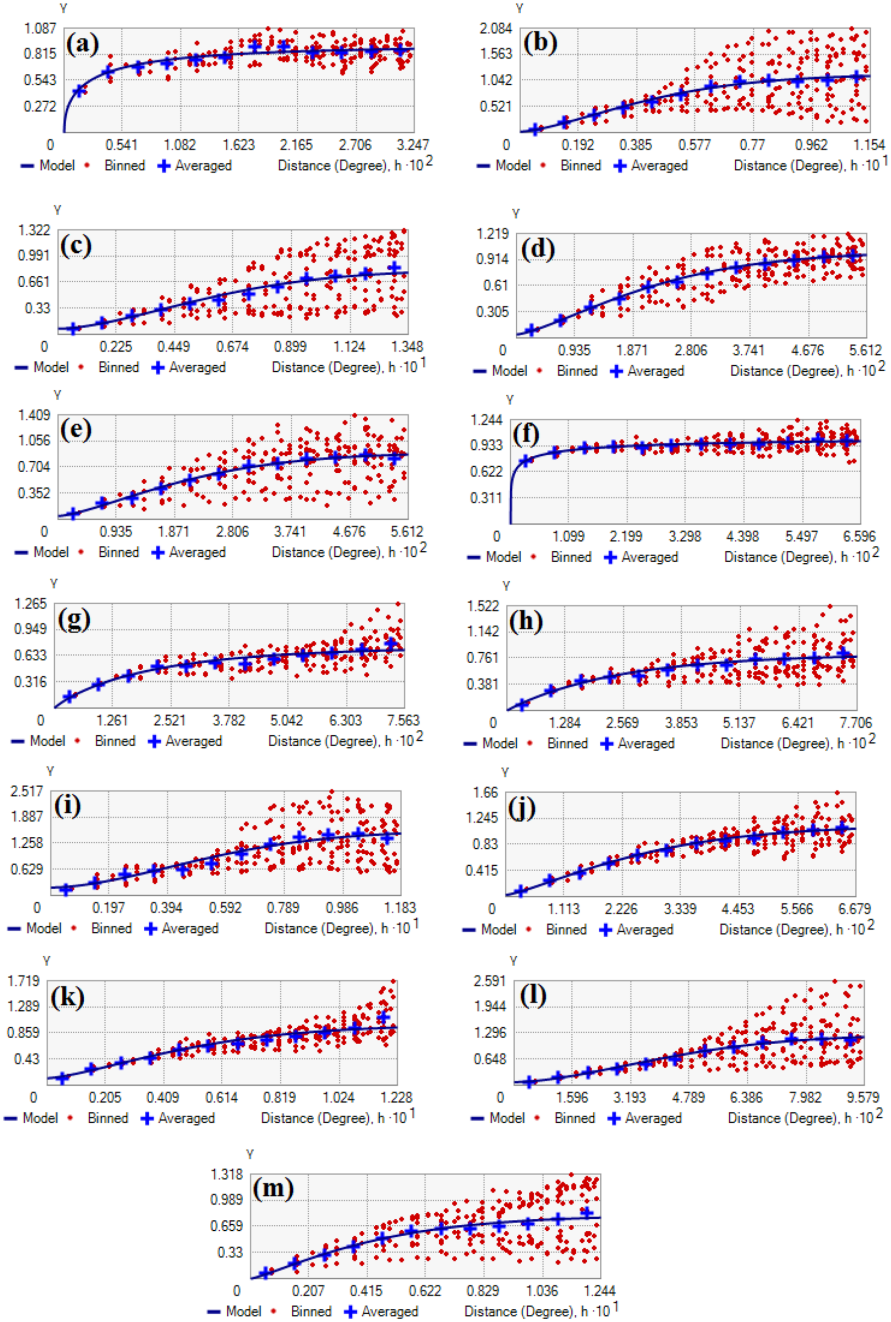
Result of semivariogram. (**a**) Distance to street, (**b**) Pressure, (**c**) Wind speed, (**d**) Humidity, (**e**) Temperature, (**f**) Distance to park, (**g**) PM 2.5, (**h**) PM 10, (**i**) SO_2_, (**j**) NO_2_, (**k**) CO, (**l**) O_3_, and (**m**) Rainfall. This map was generated using the tool of ArcGIS 10.3 (ESRI, Redlands, CA, USA, http://www.esri.com).

The results of semivariogram are shown in Fig. 5 and Table 2. According to the results of the nugget, its highest value is related to SO_2_, CO, O_3_, and wind speed, whereas its lowest value is related to the distance to street, distance to park, PM 10, PM 2.5, and rainfall. The results of the range showed that its highest value is related to the wind speed, CO, and SO_2_, whereas its lowest value is related to the distance to street, humidity, and temperature. According to the results of the sill, its highest value is related to SO_2_, pressure, and O_3_, whereas its lowest value is related to rainfall, wind speed, and PM 2.5. The results of the SD index showed that its lowest value is related to the distance to street, distance to park, PM 2.5, PM 10, and rainfall, whereas its highest value is related to SO_2_, CO, and wind speed.

**Table 2:** Results of the semivariogram parameters.

Criterion	Nugget	Range	Partial Sill	SD
Distance to street	0	0.02585	0.90347	0%
Pressure	0.020703	0.10298	1.143515	1.77%
Wind speed	0.0723	0.13484	0.746103	8.83%
Humidity	0.04172	0.053865	0.968473	4.12%
Temperature	0.04508	0.051281	0.85825	4.99%
Distance to park	0	0.06541	1.041133	0%
PM 2.5	0	0.075632	0.73868	0%
PM 10	0	0.077056	0.824453	0%
SO_2_	0.193513	0.11833	1.36949	12.38%
NO_2_	0.028775	0.066788	1.107712	2.53%
CO	0.11694	0.122836	0.877	11.76%
O_3_	0.079762	0.095789	1.17747	6.34%
Rainfall	0	0.116171	0.080033	0%

### Result of FR model

**Figure 6:**
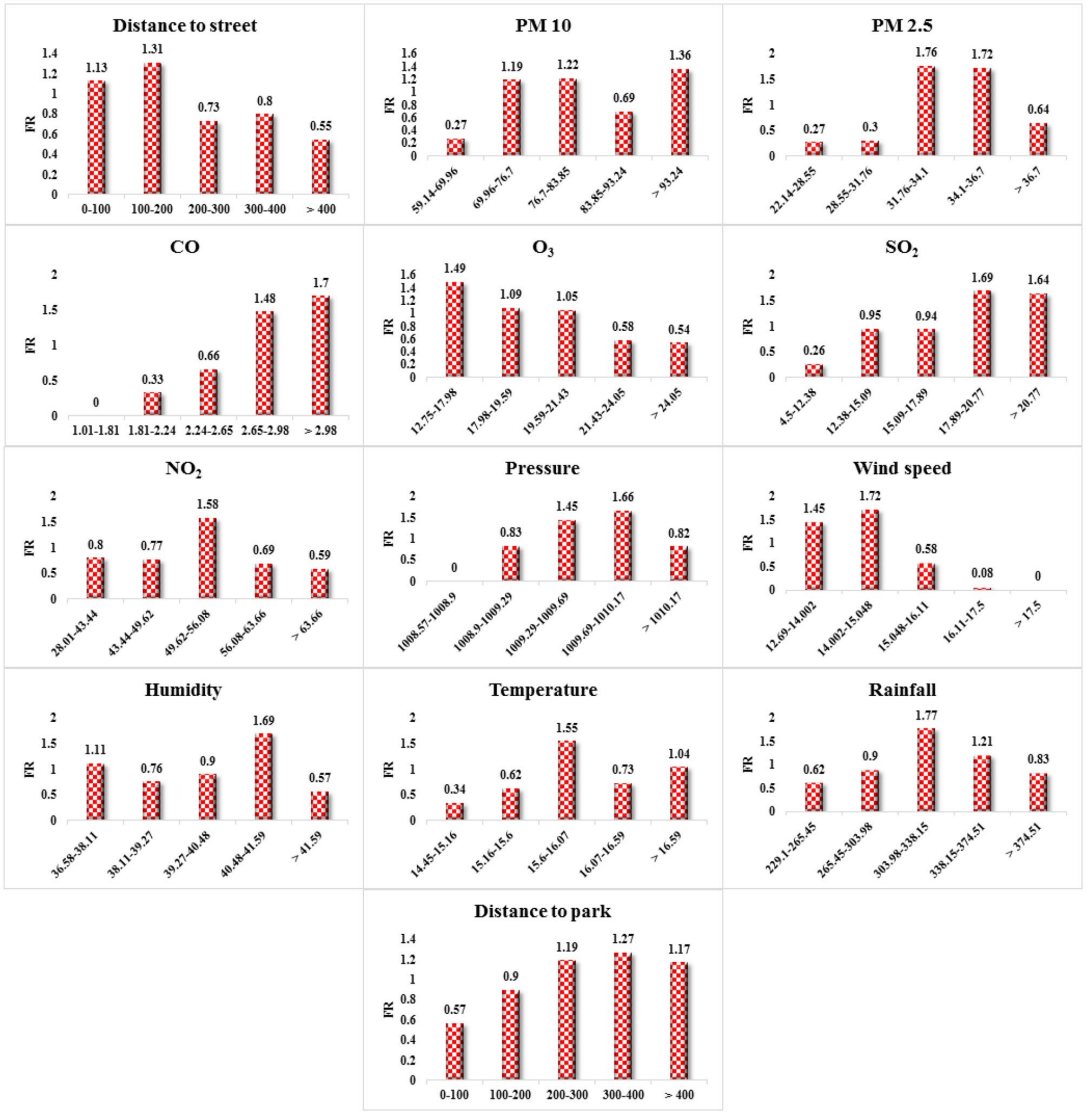
Result of frequency ratio (FR) model.

Figure 6 demonstrates the spatial relationship between asthma and the environmental criteria affecting it. According to the results of distance to street, the highest weight value of FR is related to the class 100–200 m, and also at shorter distances, there is a positive correlation between the occurrence of asthma and the distance to street. The results of the PM 10 criterion indicate that the highest FR is related to the class greater than 93.24 and the probability of asthma increases as the PM10 criterion increases. The results of PM 2.5 imply that asthma is more likely to occur in the middle classes of this criterion. According to the results of CO, as this criterion increases, the value of FR increases, thereby the probability of asthma increases. The results of O_3_ signify that the value of FR is higher in lower values of this criterion. This criterion seems to have a negative correlation with the occurrence of asthma in the study area. The results of SO_2_ show that the value of FR as well as the probability of asthma increase as the values of this criterion increase. According to the results of NO_2_, asthma is more likely to occur in the middle classes of this criterion. The results of the pressure criterion show that the highest FR value is related to the class 1009.69–1010.17 and the increase of pressure is directly related to the occurrence of asthma. The value of the FR in the wind speed criterion implies that asthma is more likely to occur in the lower values of this criterion. However, its effect on the incidence of asthma is not apparent. The results of the humidity criterion demonstrate that the highest FR value is related to the class 40.48–41.59. According to the results of the temperature criterion, the middle classes of this criterion have a higher FR value. The results of rainfall criterion denote that the highest FR is related to the class 303.98–338.15. The results of distance to parks suggest that the probability of asthma increases as the distance to parks increases, and this parameter has a positive correlation with asthma.

### Result of RF model

**Figure 7:**
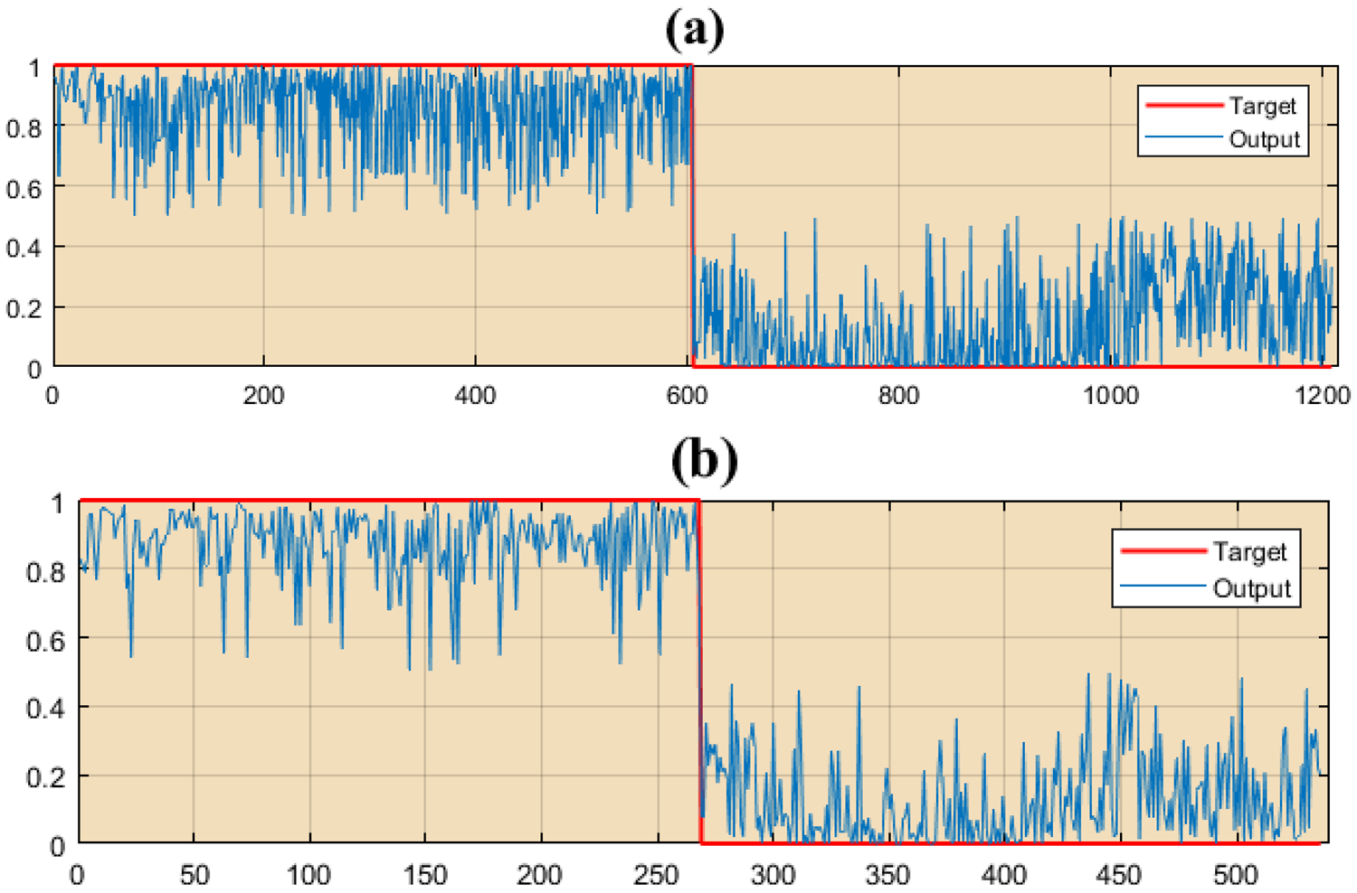
Result of random forest (RF) model. (**a**) Training data and (**b**) Validation data.

To model the asthma-prone areas using the RF model of the weights obtained, the FR model was used for each criterion and location of asthmatics. To implement the RF model, besides the places where asthma occurred, we needed places where asthma did not occur. For this purpose, the number of asthma locations (value 1) and non-asthma locations (value 0) were randomly generated and considered as target data. Spatial database including the weights obtained from the FR model for each criterion (13 environmental criteria) as well as locations of occurrence and nonoccurrence of asthma was considered for the input of RF model. From the data, 70% (604 locations of asthma patients) were used as training data and 30% (268 locations of asthma patients) as test data. RF model was implemented in the Waikato Environment for Knowledge Analysis software. The fit of the training and test data to the target data is shown in Fig. 7. The results of the RF model performance are presented in Table 3.

**Table 3:** Results of the model performance.

Metric indexes	Train	Validation
RMSE	0.236	0.347
MAE	0.1605	0.286
TP	0.934	0.864
FP	0.066	0.166
AUC	0.987	0.921

Based on the results, the values of RMSE, MAE, TP, FP, and AUC parameters are 0.236, 0.1605, 0.934, 0.066, and 0.987, respectively, for training data and 0.347, 0.286, 0.864, 0.166, and 0.921, respectively, for test data.

**Figure 8:**
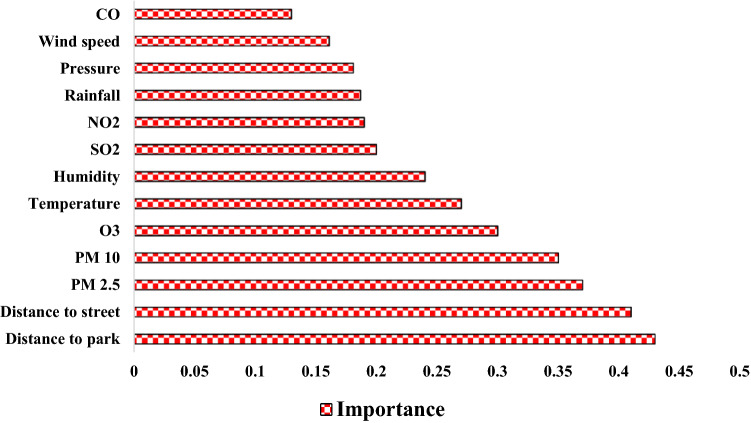
Importance of effective factors on asthma.

The importance of each effective criterion for modeling asthma-prone areas was prepared using an RF model and is shown in Fig. 8. According to the results, distance to park, distance to street, PM 2.5, and PM 10 are most important in modeling asthma-prone areas, whereas pressure, wind speed, and CO are least important.

**Figure 9:**
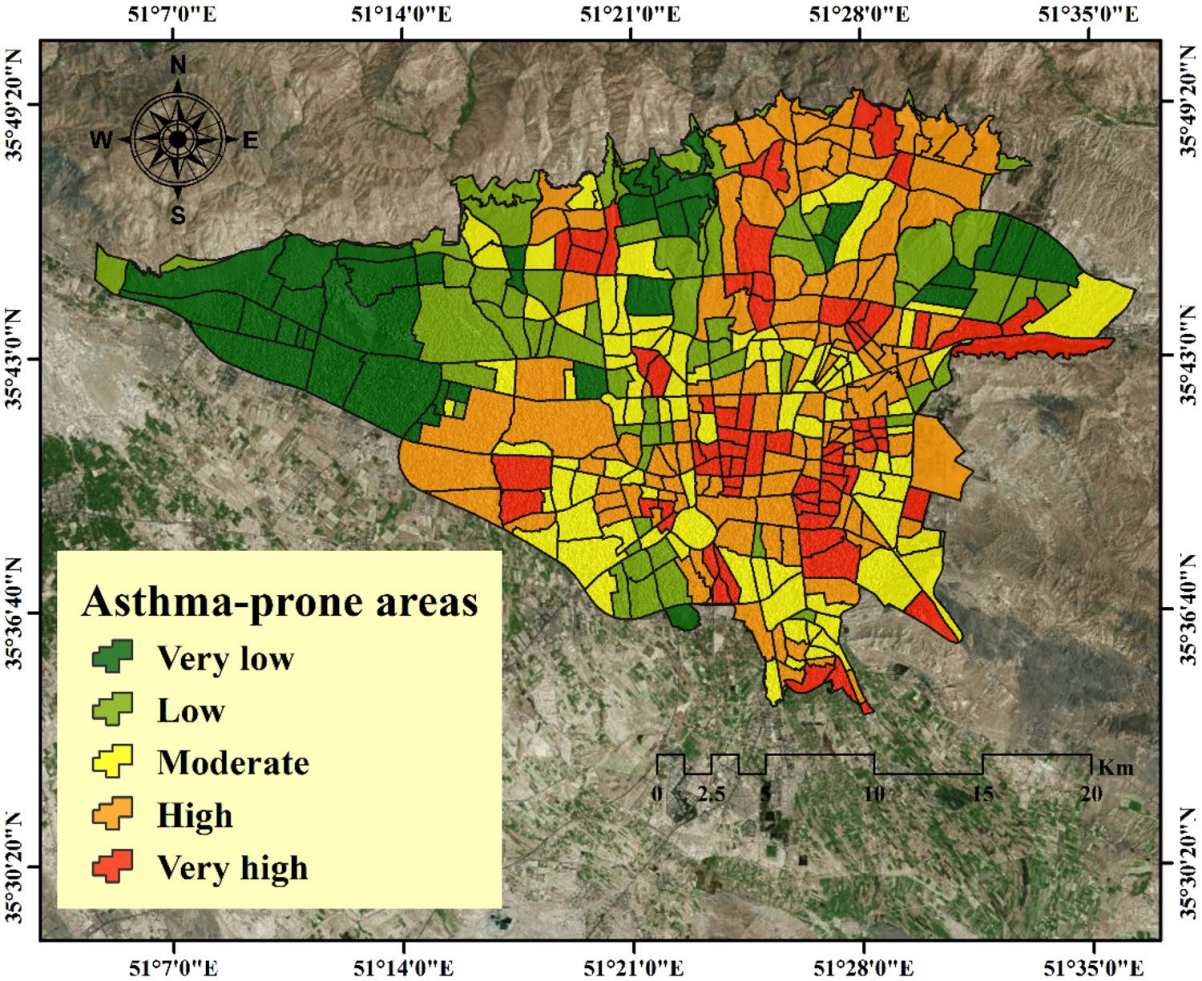
Asthma-prone areas mapping by RF model. This map was generated using the tool of ArcGIS 10.3 (ESRI, Redlands, CA, USA, http://www.esri.com).

After modeling the training data using the RF model, the fitted model was generalized to the entire study area. For this purpose, the output results were transferred to ArcGIS 10.3 software and the final map of asthma-prone areas in Tehran was prepared using an RF model. Using the Natural breaks classification method, it was divided into five classes ranging from very low risk to very high risk (see Fig. 9). According to the results, the central and southeastern regions of Tehran are more dangerous than other regions.

### Validation of final map

To evaluate the modeling results, 30% of the locations of asthmatics were used. To validate the final map, the number of asthma locations (value 1) (268 locations) and non-asthma locations (value 0) (268 locations) were randomly generated. According to them, the AUC value of the RF model in mapping asthma-prone areas is 0.987 and 0.921, respectively, for training and testing data.

**Figure 10:**
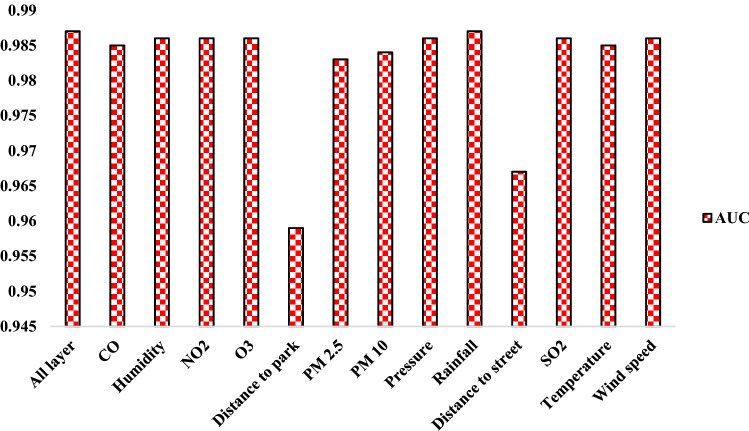
Result of RD index.

The results of sensitivity analysis using the RD index are shown in Table 4 and Fig. 10. According to them, the criteria of distance to park and distance to street are most important in modeling. These two criteria increase the modeling accuracy by 2.83% and 2.26%, respectively. The rainfall criterion is least important in modeling, thereby having no effect on the accuracy of modeling.

**Table 4:** Results of the RD index.

Excluded factor	${AUC}_{i}(\%)$	Relative decrease (RD) of AUC (%)
CO	98.5	0.202
Humidity	98.6	0.101
NO_2_	98.6	0.101
O_3_	98.6	0.101
Distance to park	95.9	2.83
PM 2.5	98.3	0.405
PM 10	98.4	0.303
Pressure	98.6	0.101
Rainfall	98.7	0
Distance to street	96.7	2.026
SO_2_	98.6	0.101
Temperature	98.5	0.202
Wind speed	98.6	0.101

## Discussion

The results of spatial autocorrelation indexes in the study area indicated that the distribution of asthma was not random and the occurrence of the disease was affected by environmental conditions. According to the results of semivariogram between the criteria affecting asthma, the criteria of distance to park, distance to street, PM 2.5, PM 10, and rainfall had the highest spatial dependence, while SO_2_, CO, and O_3_ criteria had the least spatial dependence. According to the results of the range parameter, the criteria of distance to street, temperature, and humidity had the highest spatial variability, while the criteria of wind speed, CO, and SO_2_ had the least spatial variability. The results of autocorrelation showed that all the criteria affecting asthma had a strong spatial correlation with asthma; among them, the criteria of distance to park, distance to street, PM 2.5, and PM 10 had a stronger spatial correlation.

According to the results of the FR model, asthma was more likely to occur at shorter distance to street. Based on the results of the FR model, spatial correlation, and RF model, the criterion of distance to street had a great impact on the occurrence of asthma in the study area. This is due to the traffic in the streets and the proximity of industrial centers near the streets^40^. The spatial relationship between the PM 10 criterion and the probability of asthma attacks showed that the latter increased as the former increased. As PM 2.5 increased, the FR value and likelihood of asthma attacks increases as well. Based on the results of FR, spatial autocorrelation, and RF model, among the air pollution criteria, PM 2.5 and PM 10 had a strong spatial relationship with the probability of asthma attacks in the study area. PM 2.5 and PM 10 are generally the result of fossil fuel activities, such as oil, gas and coal, vehicle traffic, metal smelting and processing, and power plants. PM 2.5 particles stay longer in the air and penetrate deeper into the lungs^41^. The results of the CO criterion showed that, as it increased, the FR value and probability of asthma attacks increased. Transportation and movement of vehicles produce and emit more than 70% of carbon monoxide. This gas interferes with the transport of oxygen in the human blood, leading to impaired cell respiration^42^. The O_3_ criterion showed that it had an inverse relationship with the FR value, i.e., in lower values of this parameter, asthma attacks were more likely to occur. Ozone gas is generated at an altitude of 30 km above the ground and enters the lower floors because of severe climate change^43^. It seems that, if there were no severe climate changes in the study area, this criterion could not play any role in modeling asthma. As the SO_2_ values increased, the rate of asthma attacks increased. Sulfur dioxide has a higher solubility in water than other pollutants, thereby having a high tendency to be absorbed in the respiratory tract when inhaled^44^. According to the results of the NO_2_ criterion, in its middle classes, the probability of asthma attacks was higher. As air pressure increased, the likelihood of asthma attacks in the study area increased. Changes in air pressure result in storms and climate change and can indirectly affect air pollutants and asthma. The results of wind speed criterion showed that it was inversely related to the FR value and incidence of asthma attacks; therefore, it was not effective in modeling asthma in the study area. Strong winds are able to disperse pollutants and increase dust; in the study area, however, this criterion did not have much effect on modeling areas prone to asthma because of the low wind speed. Humidity had an indirect effect on the occurrence of asthma attacks; by increasing this criterion, secondary pollutants such as sulfate and nitrate increased. The results of humidity criterion showed that the probability of asthma attacks in the study area was higher at a humidity of 40%. Rainfall criterion had an inverse relationship with the occurrence of asthma attacks; the concentration of pollutants and thus the associated chemical reactions in the atmosphere decreased as the rainfall increased. The results of rainfall showed that asthma was more likely to occur in the middle classes of this criterion (303–340 mm). The spatial relationship between the temperature and the occurrence of asthma showed that asthma was more likely to occur at 15 ℃. In general, as the temperature rises, photochemical reactions and ozone concentrations increase. The results of the distance to park showed that this criterion had a strong spatial relationship with the occurrence of asthma in the study area. Proximity to city parks has health benefits associated with physical activity, social cohesion, and stress reduction^6^. The results showed that the probability of asthma attacks in the study area increased as the distance to park increased.

The results showed that the RF model had good accuracy in modeling asthma in the study area. One of the advantages of the RF model was that using the average of several decision trees in the output results prevented overfitting by constructing a random subtree of features as well as a smaller tree using this subtree. There was no need for scalability in the RF model, because accuracy remained at a good level even without data scaling. Even in the absence of a large amount of data, RF model could be highly accurate^33^.

The most basic principle of fighting diseases is to change people’s lifestyles. In this regard, GIS could deliver health warnings to people at risk. By identifying where the disease is spreading, people become more aware of their surroundings and better understand safety issues. Furthermore, the identification of disease centers could reduce health costs and expenses.

## Conclusions

The purpose of this study was to map the areas prone to asthma in Tehran, Iran using an RF model. The results of the research are as follows:

1. The results of spatial autocorrelation showed that the criteria of distance to park, distance to street, PM 2.5, and PM 10 had a strong spatial correlation with asthma.
2. Based on the FR model results, the asthma in the study area occurrence was higher when distance to street equaled to 100–200 m, a PM 10 more than 93.24, a PM 2.5 between 31.76 and 34.1, a CO more than 2.98, an O_3_ between 12.75 and 17.98, an SO_2_ between 17.89 and 20.77, an NO_2_ between 49.62 and 56.08, a pressure between 1009.69 and 1010.17, a wind speed between 14.002 and 15.048, a humidity between 40.48 and 41.59, a temperature between 15.6 and 16.07, a rainfall between 303.98 and 338.15, and a distance to park between 300 and 400 m.
3. Based on the results of the RF model, the criteria of distance to park, distance to street, PM 2.5, and PM 10 had the greatest impact on the modeling of asthma areas.
4. The results showed a good accuracy (AUC is equal to 0.987 and 0.921, respectively, for training and testing) of the RF model in modeling areas prone to asthma.
5. Deploying disease risk maps using GIS could help prevent, manage, and control diseases.
